# Correlation between systemic immune inflammatory index and prognostic nutritional index and prognosis of osteosarcoma patients and construction of prediction model

**DOI:** 10.1186/s12891-026-09494-6

**Published:** 2026-01-22

**Authors:** Qingsong Huang, Jinquan Li, Xihong Sun, Zegan Sun

**Affiliations:** Department of Orthopaedics and Traumatology, 900th Hospital of PLA Joint Logistics Support Force, Fuzong Clinical Medical College of Fujian Medical University, Fuzhou, Fujian 350025 P.R. China

**Keywords:** Osteosarcoma, Systemic immune inflammatory index, Prognostic nutritional index, Nomogram, Survival analysis

## Abstract

**Background:**

To explore the predictive value of systemic immune inflammatory index (SII) and prognostic nutritional index (PNI) for the prognosis of patients with osteosarcoma, and to construct and validate an individualized survival prediction model.

**Methods:**

The clinical data of osteosarcoma patients who underwent surgery in the 900th Hospital of the Joint Logistics Support Force from January 2012 to January 2020 were retrospectively analyzed. Preoperative SII (platelets × neutrophils/lymphocytes) and PNI (albumin + 5 × lymphocytes) were calculated, and the optimal cutoff values​were determined by ROC curve. The Kaplan-Meier method was used to draw survival curves to analyze their relationship with the overall survival time of patients. Pearson correlation analysis was used to analyze the correlation between SII and PNI. Cox regression analysis was used to analyze independent prognostic factors, and the nomogram model was constructed using R software and internally validated.

**Results:**

The 5-year overall survival rate of the high SII group was significantly lower than that of the low SII group (27.7% vs. 87.0%, *p* < 0.05); the 5-year overall survival rate of the low PNI group was significantly lower than that of the high PNI group (23.1% vs. 83.6%, *p* < 0.05). SII was negatively correlated with PNI (*r*=-0.410, *P* < 0.001). The high SII + low PNI group had the worst prognosis, while the low SII + high PNI group had the best prognosis. Cox multivariate analysis showed that SII > 552.60, PNI ≤ 43.38, Enneking stage III, local recurrence, and metastasis were independent risk factors affecting the prognosis of osteosarcoma patients (*P* < 0.05). A nomogram for predicting the overall survival (OS) of osteosarcoma patients was constructed based on SII, PNI, Enneking stage, local recurrence, and metastasis. The C index of the model was 0.870, and the validation C index was 0.828. The calibration curve showed that the predicted and actual survival rates were highly consistent. The ROC curve shows that the AUC (0.976) of the nomogram prediction model is significantly higher than the indexes such as SII(AUC = 0.811), PNI(AUC = 0.791) and Enneking stage (AUC = 0.761).

**Conclusions:**

SII and PNI are effective biomarkers for the prognosis of osteosarcoma. The nomogram integrating these indices with clinical characteristics better reflects osteosarcoma’s multi-faceted features, providing a stronger basis for individualized treatment.

## Introduction

Osteosarcoma is the most common primary malignant bone tumor, which is prone to occur in adolescents. It has a high degree of malignancy and mortality, resulting in a poor prognosis for patients. The 5-year survival rate is about 50–70%, which seriously threatens the life safety of patients [1, 2]. In addition, osteosarcoma is prone to early metastasis. Although neoadjuvant chemotherapy combined with limb-saving surgery has significantly improved the local control rate, 30%-40% of patients still have distant metastasis, and the survival period of osteosarcoma patients with distant metastasis is often less than 5 years [3]. Therefore, strengthening the prognosis assessment of osteosarcoma patients is the focus of current clinical work. As the main method to evaluate the prognosis of osteosarcoma patients in clinic, Enneking staging system can effectively reflect the anatomical invasion range of the tumor, but its core limitation is that it does not include the tumor microenvironment characteristics of the host, which has been proved to be the key factor affecting the tumor progress and prognosis [4]. Imbalance of immune inflammation in tumor microenvironment (e.g., inflammatory reaction that promotes cancer caused by abnormal infiltration of neutrophils and platelets) and deficiency of nutritional-immune function (e.g., decrease of anti-tumor immunity caused by low albumin and lymphopenia) directly affect the prognosis of patients by regulating tumor cell proliferation, angiogenesis and immune escape [5–8]. In clinical practice, it is difficult to comprehensively evaluate these dynamic host-tumor interactions only by Enneking staging, which may lead to the deviation of prognosis evaluation. Therefore, exploring the systemic immune inflammatory index (SII) and the prognostic nutritional index (PNI) which can quantitatively reflect the balance of immune inflammation is expected to make up for the deficiency of traditional staging in microenvironment assessment and provide a more comprehensive biological basis for the prognosis assessment of osteosarcoma.

The systemic immune inflammatory index (SII), which integrates platelet, neutrophil, and lymphocyte counts, comprehensively reflects the balance between pro-tumor and anti-tumor immunity. Its prognostic value has been validated in multiple malignancies: in non-metastatic renal cell carcinoma, Hu et al. [9] found that high level of SII and poor overall survival (OS) (HR = 2.26; 95%CI: 1.44–3.54; *P* < 0.001) and cancer-specific survival (CSS) (HR = 2.17; 95%CI: 1.33–3.55; *P* = 0.002). Similarly, in gastric cancer, Cao et al. [10] also confirmed that high SII is obviously related to poor prognosis by searching related literature. On the other hand, the prognostic nutritional index (PNI), which combines serum albumin and lymphocyte count, reflects both nutritional reserve and immune status. In gastric cancer, Nogueiro et al. [11] reported that compared with the high PNI group, the OS of patients in the low PNI group was significantly shortened (40.26 vs. 77.49 months; *p* < 0.001). For patients with non-small cell lung cancer (NSCLC), Xu et al. [12] proved that PNI was an independent prognostic factor (*P* = 0.002), and the 2-year OS of patients with low PNI was significantly lower than that of patients with high PNI (10.4% vs. 26.7%, *P* < 0.05). SII and PNI, though measuring distinct biological processes, are inherently linked through this “inflammation-nutrition-immunity” axis. SII quantifies the pro-tumorigenic imbalance between pro-inflammatory cells (neutrophils, platelets) and anti-tumor immune cells (lymphocytes), while PNI integrates nutritional reserves (albumin) and immune competence (lymphocytes). Their combination thus provides a more comprehensive assessment of the host’s systemic response to tumors than either index alone. Previous studies in other malignancies (e.g., colorectal cancer, hepatocellular carcinoma) have shown that the synergistic analysis of inflammatory and nutritional-immune markers improves prognostic accuracy [13, 14]. However, in osteosarcoma, there is still little evidence about the predictive value of SII and PNI. Based on this, this study aims to analyze and explore the correlation between preoperative SII, PNI and the prognosis of osteosarcoma patients, and innovatively combine SII and PNI to construct a prediction model to facilitate better individualized treatment.

## Materials and methods

### Data collection

The clinical data of patients with osteosarcoma who underwent surgical treatment in the 900th Hospital of the Joint Logistics Support Force from January 2012 to January 2020 were collected. Inclusion criteria: (1) postoperative pathological diagnosis of osteosarcoma; (2) no previous anticancer treatment; (3) complete medical records and follow-up data; (4) no distant metastasis or unresectable tumor at the initial diagnosis. Exclusion criteria: (1) preoperative complications of acute and chronic inflammation; (2) preoperative complications of blood system diseases; (3) preoperative use of nonsteroidal anti-inflammatory drugs; (4) missing medical records or lost to follow-up; (5) refusal of surgical treatment. All included patients underwent curative radical resection with the goal of complete tumor removal. The surgical procedures primarily included limb-salvage surgery (for extremity tumors) or radical resection of the primary tumor (for non-extremity tumors), with clear surgical margins confirmed by intraoperative frozen pathology and postoperative histopathological examination. No palliative procedures (e.g., tumor debulking, bypass surgery) were included. This study was approved by the Ethics Committee of the 900th Hospital of the Joint Logistics Support Force, and the ethical approval number is 2025-051. All patients or their legal guardians provided written informed consent for the use of their clinical data in this study.

### Analytical method

Clinical parameters including age, gender, tumor site, tumor size, histological type, Enneking stage, pathological fracture, local recurrence, metastasis, neoadjuvant chemotherapy, postoperative radiotherapy, postoperative chemotherapy, laboratory data (platelet, neutrophil, lymphocyte, albumin) were collected. SII, PLR, NLR, and PNI were calculated. Laboratory data (platelet, neutrophil, lymphocyte, albumin) were collected from venous blood samples within 1 week before the initial surgery, with no prior anticancer treatment or perioperative interventions that might affect these indicators. SII = peripheral platelet (10^9^ /L) × neutrophil (10^9^ /L) / lymphocyte count (10^9^ /L), PLR = peripheral platelet (10^9^ /L) / lymphocyte count (10^9^ /L), NLR = neutrophil (10^9^ /L) / lymphocyte count (10^9^ /L), PNI = serum albumin + 5 × lymphocyte count (10^9^ /L). The patients were grouped according to the optimal critical value, and the relationship between SII, PNI and clinical pathological factors of the patients was analyzed.

### Follow-up

All patients were followed up regularly after surgery, and we contacted the patients through outpatient examinations, telephone or letters. Physical examination, blood test, and chest X-ray examination are routine clinical evaluations in our hospital. The follow-up deadline is March 1, 2025. OS is defined as the time from surgery to tumor-related death or the end of follow-up.

### Statistical analysis

SPSS 26.0 and R (4.4.0) software were used for statistical analysis. Count data were expressed as n (%), and intergroup comparisons were performed using the χ^2^ test. Receiver operating characteristic curve (ROC curve) analysis was used to determine the optimal critical values​of SII, PNI, PLR, and NLR. Survival analysis was performed using the Kaplan-Meier method. Univariate and multivariate analyses were performed using the Cox proportional hazard regression model to identify the factors affecting the prognosis of patients with osteosarcoma, and the hazard ratio (HR) and 95% confidence interval (CI) were used to evaluate the relative risk. ROC curve was used to evaluate the predictive power of nomogram, SII, PNI and other indicators in predicting the prognosis of osteosarcoma patients. Pearson correlation analysis was used to analyze the correlation between SII and PNI. Furthermore, to verify potential collinearity among variables in the multivariate analysis—particularly between SII (which includes lymphocytes) and PNI (which includes lymphocytes)—this study performed a variance inflation factor (VIF) test. The VIF values of all variables included in the multivariate Cox regression model (SII, PNI, Enneking stage, local recurrence, and metastasis) were calculated. A VIF > 5 was generally considered indicative of significant collinearity. Additionally, to further validate the stability of variable selection, a sensitivity analysis using LASSO regression (implemented via the ‘glmnet’ package in R) was conducted. Variables corresponding to the λ.min value were retained as final factors in the model to ensure the reliability of the results. According to the results of Cox multivariate analysis, the “rms” package in R software (version 3.6.0) was used to establish a nomogram prediction model. In the construction of the nomogram, continuous variables SII and PNI were binned into binary categories based on their optimal cutoff values (SII = 552.60, PNI = 43.38) determined by ROC curve analysis with the maximum Youden index, enhancing clinical interpretability; the score assignment for each variable was derived from the β-coefficients of the multivariate Cox regression model, with absolute values of β-coefficients standardized to a 0−100 scale and total scores mapped to 1-, 3-, and 5-year OS probabilities; for internal validation, Bootstrap resampling with 1000 iterations was performed without training-validation set split (to avoid reducing statistical power in the moderate sample size of 135), with each iteration sampling 135 patients with replacement, reconstructing the model, and calculating the C-index, and the validation C-index (0.828) was determined as the median across iterations to reflect robustness. The Harrell consistency index (C-index) was used to quantify the discriminative performance of the prediction nomogram. The larger the C-index, the more accurate the prognosis prediction. *P* < 0.05 was considered statistically significant.

## Results

### General data

A total of 135 patients met the inclusion criteria, including 73 males (54.1%) and 62 females (45.9%), with an average age of 20.9 ± 5.1 (range: 7–33 years). All 135 patients were diagnosed with osteosarcoma, 106 patients (78.5%) had tumors located in the limbs, and 29 patients (21.5%) had tumors not located in the limbs; the average tumor diameter was 4.2 ± 0.9 cm. There were 59 patients (43.7%) with well-differentiated and 76 patients (56.3%) with poorly differentiated tumors, respectively.

### Determination of the optimal critical values of SII, PNI, PLR and NLR before surgery

**Fig. 1:**
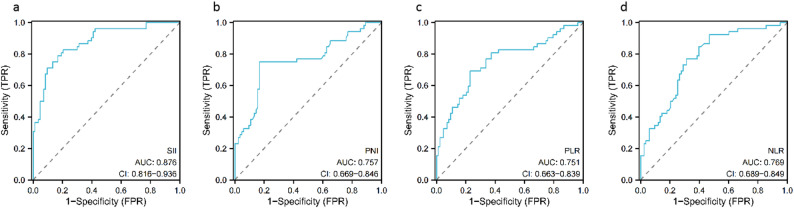
ROC curve of preoperative SII, PNI, PLR, and NLR for predicting overall survival of patients with osteosarcoma. **a**: SII, **b**: PNI, **c**: PLR, **d**: NLR

The ROC curves of SII, PNI, PLR, and NLR were drawn and the corresponding optimal critical values​were determined. The value corresponding to the maximum Youden index was taken as the optimal critical value, where SII was 552.60, PNI was 43.38, PLR was 187.70, and NLR was 2.94. According to this cutoff value, the patients were divided into two groups for further analysis: low SII group (SII ≤ 552.60, *n* = 75) and high SII group (SII > 552.60, *n* = 60), low PNI group (PNI ≤ 43.38, *n* = 53) and high PNI group (PNI > 43.38, *n* = 82), low PLR group (PLR ≤ 187.70, *n* = 80) and high PLR group (PLR > 187.70, *n* = 55), low NLR group (NLR ≤ 2.94, *n* = 69) and high NLR group (NLR > 2.94, *n* = 66), as shown in Fig. 1.

### Relationship between preoperative SII, PNI and clinicopathological characteristics of patients with osteosarcoma

By analyzing the relationship between SII, PNI and the clinical characteristics of osteosarcoma, we found that SII was correlated with Enneking stage, local recurrence, metastasis, PLR and NLR, and the difference was statistically significant (*P* < 0.05), but was not related to age, gender, tumor location, tumor size, degree of differentiation, pathological fracture, and neoadjuvant chemotherapy, and the difference was not statistically significant (*P* > 0.05); PNI was correlated with tumor size, Enneking stage, metastasis, PLR and NLR, and the difference was statistically significant (*P* < 0.05), but was not related to age, gender, tumor location, degree of differentiation, pathological fracture, local recurrence, and neoadjuvant chemotherapy, and the difference was not statistically significant (*P* > 0.05), as shown in Table 1.

**Table 1:** Relationship between SII, PNI and clinicopathological factors in patients with osteosarcoma[n(%)]

Index	Low-SII (*n* = 75)	High-SII (*n* = 60)	χ^2^	*P*	Low-PNI (*n* = 53)	High-PNI (*n* = 82)	χ^2^	*P*
Age (years)			0.452	0.501			0.877	0.349
≤ 20	27 (36.0)	25 (41.7)			23 (43.4)	29 (35.4)		
> 20	48 (64.0)	35 (58.3)			30 (56.6)	53 (64.6)		
Gender			0.037	0.847			1.396	0.237
Male	40 (53.3)	33 (55.0)			32 (60.4)	41 (50.0)		
Female	35 (46.7)	27 (45.0)			21 (39.6)	41 (50.0)		
Tumor location			0.141	0.708			2.110	0.146
Limbs	58 (77.3)	48 (80.0)			45 (84.9)	61 (74.4)		
Non-limbs	17 (22.7)	12 (20.0)			8 (15.1)	21 (25.6)		
Tumor size (cm)			1.436	0.231			5.680	0.017
≤ 5	57 (76.0)	40 (66.7)			32 (60.4)	65 (79.3)		
> 5	18 (24.0)	20 (33.3)			21 (39.6)	17 (20.7)		
Degree of differentiation			0.182	0.670			0.003	0.954
Well differentiated	34 (45.3)	25 (41.7)			23 (43.4)	36 (43.9)		
Poorly differentiated	41 (54.7)	35 (58.3)			30 (56.6)	46 (56.1)		
Enneking staging			13.500	< 0.001			12.177	< 0.001
Stage I, II	60 (80.0)	30 (50.0)			26 (49.1)	64 (78.0)		
Stage III	15 (20.0)	30 (50.0)			27 (50.9)	18 (20.0)		
Pathological fracture			0.448	0.503			0.001	0.971
No	54 (72.0)	40 (66.7)			37 (69.8)	57 (69.5)		
Yes	21 (28.0)	20 (33.3)			16 (30.2)	25 (30.5)		
Local recurrence			3.921	0.048			1.272	0.259
No	67 (89.3)	46 (76.7)			42 (79.2)	71 (86.6)		
Yes	8 (10/7)	14 (23.3)			11 (20.8)	11 (13.4)		
Metastasis			24.368	< 0.001			12.116	< 0.001
No	71 (94.7)	36 (60.0)			34 (64.2)	73 (89.0)		
Yes	4 (5.3)	24 (40.0)			19 (35.8)	9 (11.0)		
Neoadjuvant chemotherapy			0.015	0.904			2.004	0.157
No	48 (64.0)	39 (65.0)			38 (71.7)	49 (59.8)		
Yes	27 (36.0)	21 (35.0)			15 (28.3)	33 (40.2)		
Postoperative radiotherapy			0.042	0.839			0.937	0.333
No	72 (96.0)	58 (96.7)			50 (94.3)	80 (97.6)		
Yes	3 (4.0)	2 (3.3)			3 (5.7)	2 (2.4)		
Postoperative chemotherapy			3.000	0.083			2.516	0.113
No	19 (25.3)	8 (13.3)			7 (13.2)	20 (24.4)		
Yes	56 (74.7)	52 (86.7)			46 (86.8)	62 (75.6)		
PLR			57.737	< 0.001			19.807	< 0.001
≤ 187.70	66 (88.0)	14 (23.3)			19 (35.8)	61 (74.4)		
> 187.70	9 ( 12.0)	46 (76.7)			34 (64.2)	21 (25.6)		
NLR			73.049	< 0.001			15.286	< 0.001
≤ 2.94	63 (84.0)	6 (10.0)			16 (30.2)	53 (64.6)		
> 2.94	12 (16.0)	54 (90.0)			37 (69.8)	29 (35.4)		

### Relationship between preoperative SII, PNI, PLR, NLR and overall survival in patients with osteosarcoma

**Fig. 2:**
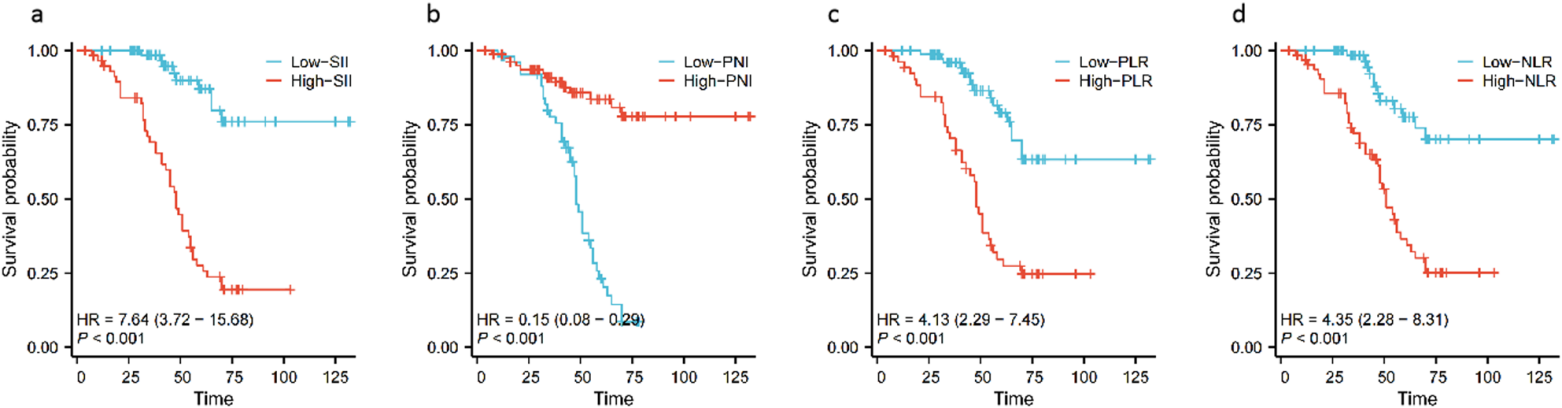
Overall survival curve of two groups of osteosarcoma patients after surgery. **a**: SII, **b**: PNI, **c**: PLR, **d**: NLR

The median follow-up time was 68.5 months. The 1-, 3-, and 5-year survival rates of the low SII group were 100.0%, 98.5%, and 87.0%, respectively, and the 1-, 3-, and 5-year survival rates of the high SII group were 96.6%, 69.1%, and 27.7%, respectively. The OS of the high SII group was significantly lower than that of the low SII group, and the difference in overall survival between the two groups was statistically significant (χ^2^=42.682, *P* < 0.05) (Fig. 2a). The 1-, 3-, and 5-year survival rates of the low PNI group were 98.1%, 79.8%, and 23.1%, respectively; the 1-, 3-, and 5-year survival rates of the high PNI group were 98.8%, 90.9%, and 83.6%, respectively. The OS of the high PNI group was significantly higher than that of the low PNI group, and the difference in overall survival between the two groups was statistically significant (χ^2^=42.587, *P* < 0.05 ) (Fig. 2b); the 1-, 3-, and 5-year survival rates of the low PLR group were 100.0%, 95.9%, and 78.9%, respectively, and those of the high PLR group were 96.2%, 70.4%, and 29.8%, respectively. The OS of the high PLR group was significantly lower than that of the low PLR group, and the difference in overall survival rate between the two groups was statistically significant (χ^2^=26.252, *P* < 0.05) (Fig. 2c); the 1-, 3-, and 5-year survival rates of the low NLR group were 100.0%, 98.4%, and 77.4%, respectively, and those of the high NLR group were 96.9%, 72.1%, and 36.6%, respectively. The OS of the high NLR group was significantly lower than that of the low NLR group, and the difference in overall survival rate between the two groups was statistically significant (χ^2^=23.840, *P* < 0.05) (Fig. 2d).

### Univariate and multivariate Cox analysis of factors affecting overall survival in patients with osteosarcoma

**Fig. 3:**
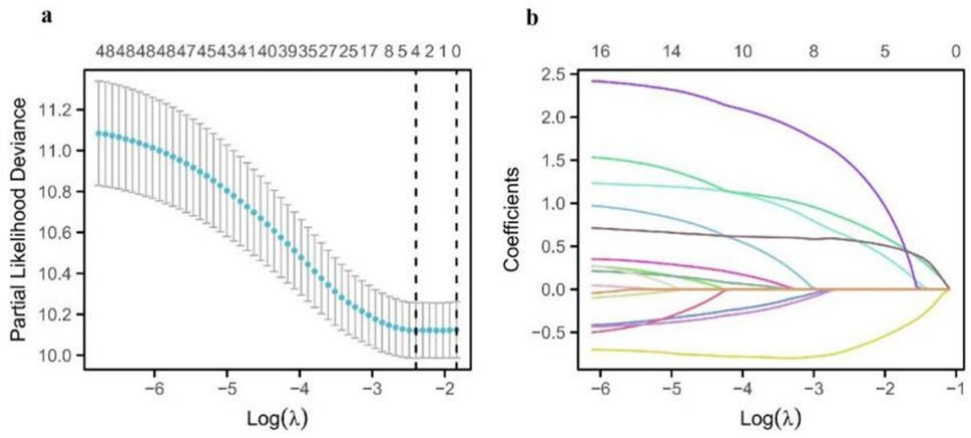
Screening prognostic risk factors of patients with osteosarcoma by LASSO analysis. **a**: the relation curve between partial likelihood deviance and log (λ) is drawn. **b**: LASSO coefficient distribution of 16 characteristic factors, in which the best λ produces 5 features with non-zero coefficients

**Fig. 4:**
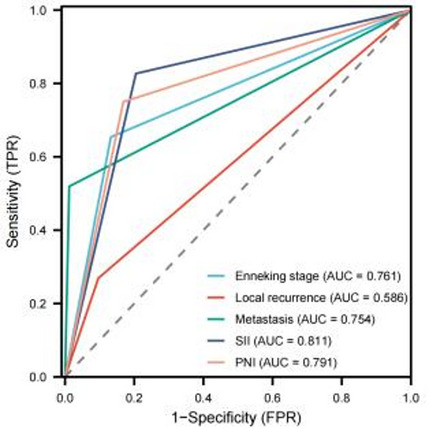
ROC curve of Enneking staging, local recurrence, metastasis, SII and PNI in predicting the prognosis of osteosarcoma patients

We further performed univariate Cox regression analysis, and the results showed that tumor size, Enneking stage, local recurrence, metastasis, SII, PNI, PLR, and NLR were influencing factors for the prognosis of osteosarcoma patients (*P* < 0.05). Prior to multivariate Cox regression analysis, collinearity among variables was evaluated using the VIF test: the VIF value for SII was 1.428, and for PNI was 1.268. The VIF values for Enneking stage, local recurrence, and metastasis were 1.211, 1.036, and 1.313, respectively. All VIF values were < 5, indicating no significant collinearity, and thus all variables could be included in the model simultaneously. The factors with statistical significance in the univariate analysis results were included in the multivariate Cox regression model, and the results showed that Enneking stage, local recurrence, metastasis, SII, and PNI were independent risk factors affecting the prognosis of osteosarcoma patients (*P* < 0.05), as shown in Table 2. Results of the LASSO regression sensitivity analysis showed that when the penalty coefficient λ was set to the minimum value, SII, PNI, Enneking stage, local recurrence, and metastasis were all retained (non-zero coefficients), consistent with the results of the multivariate Cox regression. This further confirmed that these variables could be included in the model as independent prognostic factors, as shown in Fig. 3. We used ROC curve to evaluate the predictive power of Enneking stage, local recurrence, metastasis, SII and PNI in osteosarcoma patients. The results showed that the AUC of SII was the highest (0.811), followed by PNI (0.791), and the AUC of Enneking stage was second only to SII and PNI, with 0.761, and that of metastasis was 0.754, while the predictive power of local recurrence was 0.754, as shown in Fig. 4.

**Table 2:** Univariate and multivariate Cox regression analysis of factors affecting the survival of patients with osteosarcoma

Index	Univariate analysis		Multivariate analysis	
	HR(95%CI)	*P*	HR(95%CI)	*P*
Age (≤ 20 vs. > 20)	0.626 (0.363 ~ 1.078)	0.091		
Gender (male vs. female)	0.705 (0.405 ~ 1.230)	0.219		
Tumor location (extremity vs. nonextremity)	0.827 (0.403 ~ 1.698)	0.605		
Tumor size (≤ 5 cm vs. > 5 cm)	2.182 (1.264 ~ 3.768)	0.005	1.151 (0.581–2.283)	0.687
Degree of differentiation (well-differentiated vs. poorly differentiated)	1.044 (0.596 ~ 1.830)	0.880		
Enneking staging (I, II vs. III)	4.652 (2.615 ~ 8.276)	< 0.001	2.869 (1.377 ~ 5.980)	0.005
SII (≤ 552.60 vs. > 552.60)	7.568 (3.685 ~ 15.541)	< 0.001	3.882 (1.452 ~ 10.381)	0.007
PNI (≤ 43.38 vs. > 43.38)	0.154 (0.081 ~ 0.292)	< 0.001	0.403 (0.183 ~ 0.886)	0.024
PLR (≤ 187.70 vs. > 187.70)	4.109 (2.278 ~ 7.412)	< 0.001	1.287 (0.587 ~ 2.819)	0.529
NLR (≤ 2.94 vs. > 2.94)	4.329 (2.269 ~ 8.258)	< 0.001	0.750 (0.316 ~ 1.779)	0.514
Pathological fracture (no vs. yes)	1.507 (0.824 ~ 2.756)	0.183		
Local recurrence (no vs. yes)	5.667 (2.997 ~ 10.718)	< 0.001	9.685 (4.259 ~ 22.023)	< 0.001
Metastasis (No vs. Yes)	5.607 (3.205 ~ 9.808)	< 0.001	1.989 (1.022 ~ 3.872)	0.043
Neoadjuvant chemotherapy (No vs. Yes)	1.235 (0.697 ~ 2.188)	0.470		
Postoperative radiotherapy (No vs. Yes)	3.360 (0.425 ~ 26.539)	0.250		
Postoperative chemotherapy (No vs. Yes)	2.617 (1.041 ~ 6.580)	0.041	0.879 (0.286 ~ 2.696)	0.821

### The prediction effect of the nomogram prediction model on the cumulative survival rate of patients with osteosarcoma

**Fig. 5:**
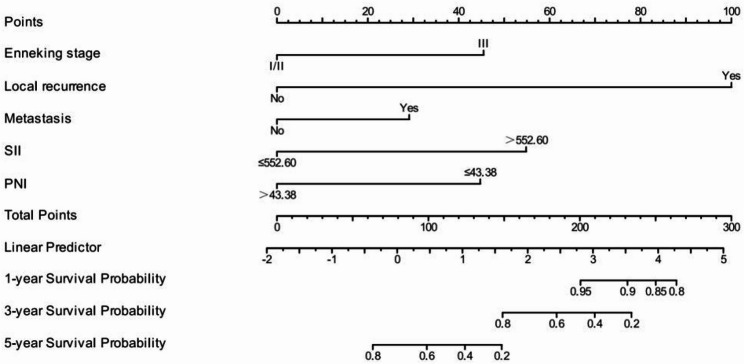
Nomogram prediction results of 1-, 3-, and 5-year survival rates in patients with osteosarcoma

**Fig. 6:**
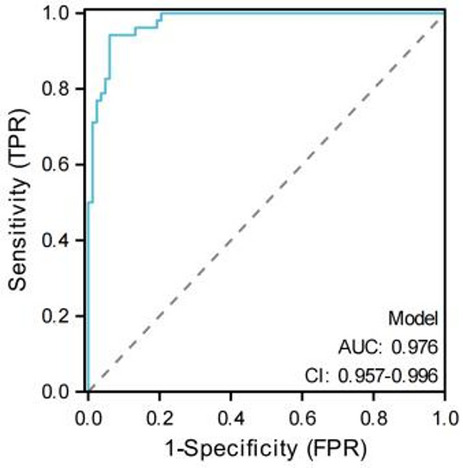
ROC curve for predicting survival rate of osteosarcoma patients by nomogram

**Fig. 7:**
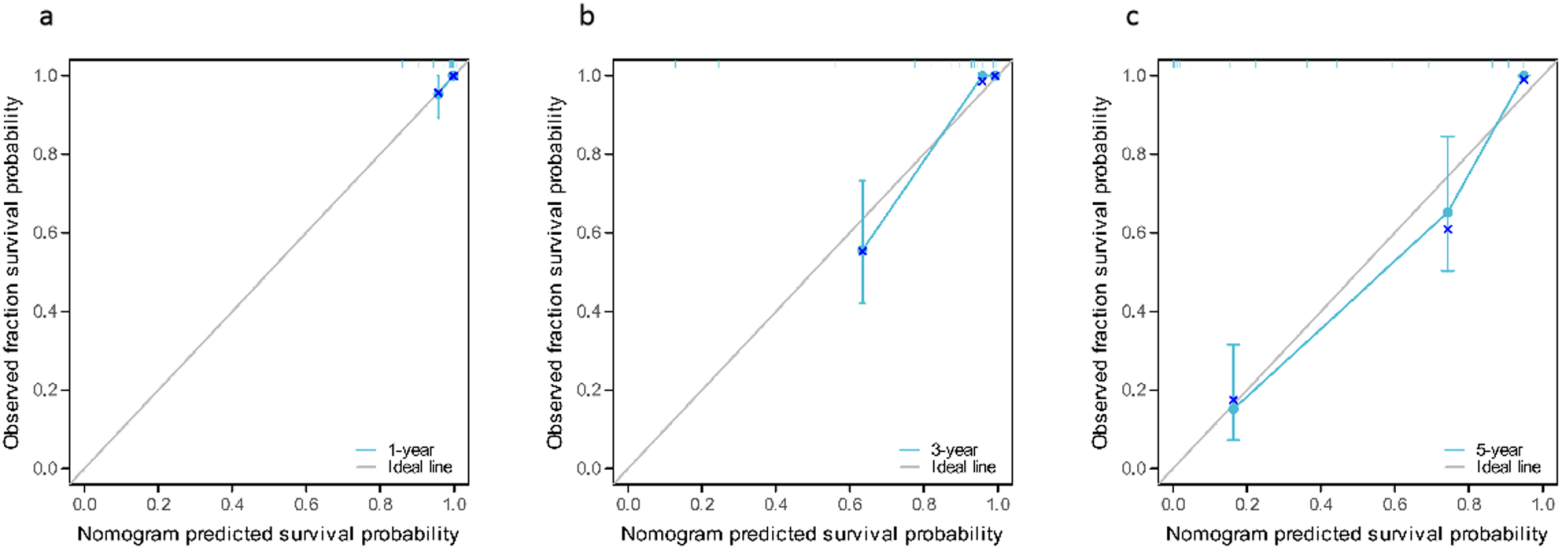
Calibration curve of the nomogram prediction model of osteosarcoma patients (modeling group). **a**: 1-year; **b**: 3-year; **c**: 5-year

**Fig. 8:**
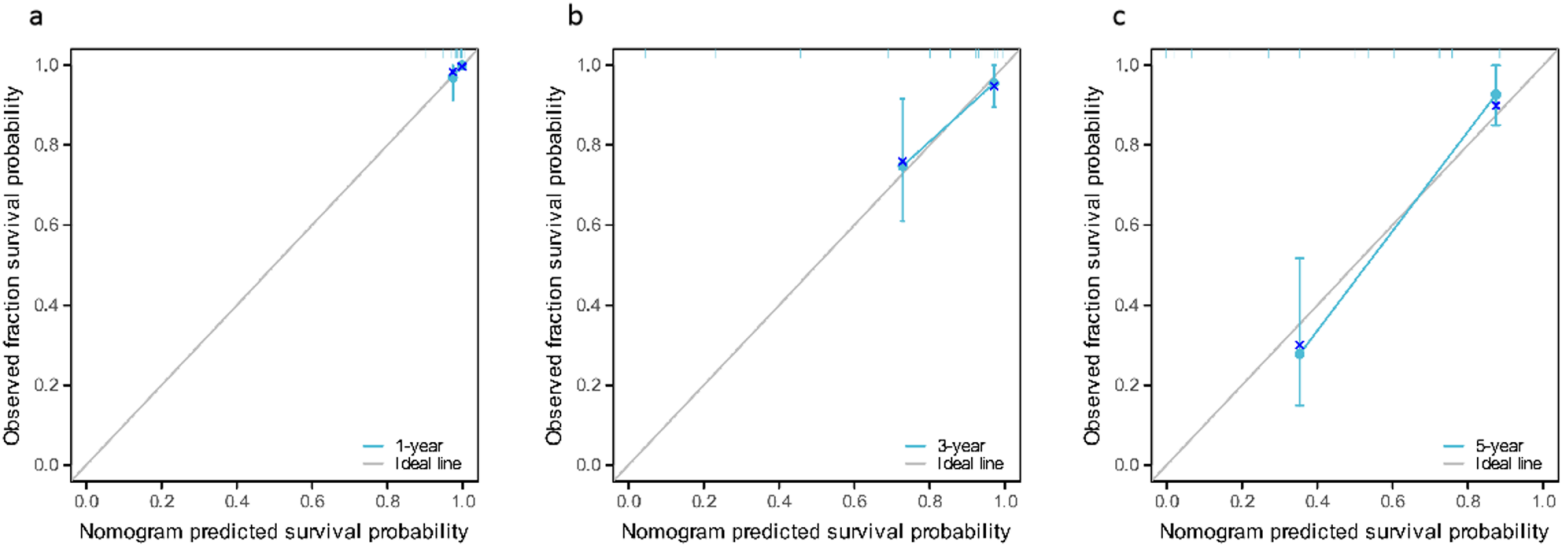
Calibration curve of the nomogram prediction model of osteosarcoma patients (validation group). **a**: 1-year; **b**: 3-year; **c**: 5-year

According to the independent risk factors screened by Cox regression model (Enneking stage, local recurrence, metastasis, SII, PNI), the model exhibited excellent discriminative ability with a C-index of 0.870(95%CI: 0.849–0.891) and maintained good performance in internal validation via 1000 Bootstrap resamplings (validation C-index = 0.828, 95%CI: 0.791–0.866). The ROC curve analysis confirmed that the nomogram had a significantly higher AUC (0.976) than individual indicators such as SII (0.811), PNI (0.791), and Enneking stage (0.761), indicating its superior predictive value for individualized prognosis assessment in osteosarcoma patients (Figs. 5 and 6), and the calibration curves demonstrated high consistency between predicted and actual OS rates, as shown in Figs. 7 and 8.

### Interaction effect between SII and PNI on prognosis

**Fig. 9:**
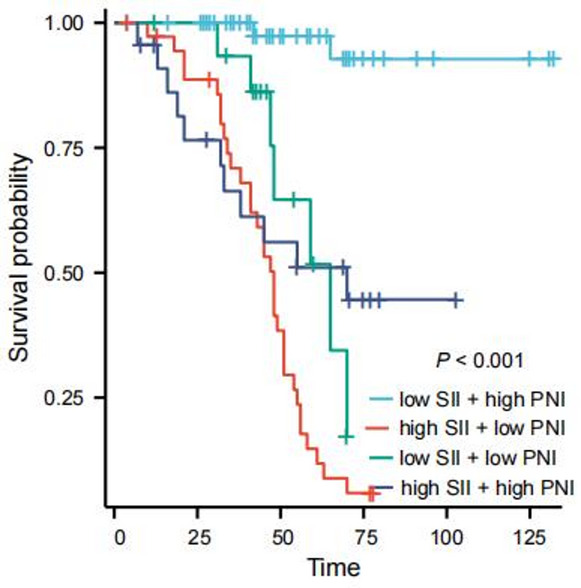
Kaplan-Meier survival curves for overall survival (OS) stratified by combinations of preoperative SII and PNI in osteosarcoma patients

To explore the interaction between SII and PNI, we used pearson analysis, and found that there was a significant negative correlation between SII and PNI (*r*=-0.410, *P* < 0.001). In addition, we divided the patients into four groups based on their cutoff values: (1) high SII + low PNI (*n* = 37); (2) high SII + high PNI (*n* = 23); (3) low SII + low PNI (*n* = 16); (4) low SII + high PNI (*n* = 59). Kaplan-Meier survival analysis showed that the 5-year OS rates of the four groups were 14.8%, 51.0%, 51.7%, and 97.4%, respectively (χ²=61.933, *P* < 0.001; Fig. 9). The high SII + low PNI group had the worst prognosis, while the low SII + high PNI group had the best prognosis, indicating a synergistic effect between SII and PNI.

## Discussion

Tumor-related inflammatory response plays a dual role in malignant transformation, progression and immune regulation. On the one hand, tumor-driving gene mutations can directly activate pro-inflammatory signaling pathways (such as NF-κB and STAT3), triggering local inflammatory cascade reactions; on the other hand, inflammatory cells such as neutrophils and macrophages recruited to the tumor microenvironment secrete cytokines such as IL-6 and TNF-α to form a pro-oncogenic inflammatory microenvironment, accelerating tumor invasion and metastasis [15, 16]. In terms of clinical prognosis evaluation, Cao Linwei et al. [17] based on a retrospective cohort study of 51 osteosarcoma patients confirmed that preoperative high neutrophil/lymphocyte ratio (NLR > 3.35) and high platelet/lymphocyte ratio (PLR > 124.91) were both associated with the prognosis of osteosarcoma patients, and NLR > 3.35 could be used as an independent risk factor for patient prognosis. In recent years, some new inflammatory indicators have emerged, such as SII and PNI. SII is a comprehensive indicator that integrates three key parameters of neutrophils, platelets and lymphocytes, while PNI is a comprehensive indicator of nutrition-immune function. The value of both in predicting the prognosis of patients with various tumors has been confirmed [18]. However, the value of SII and PNI in predicting the prognosis of osteosarcoma has not been fully elucidated, and there are few related research reports. This study evaluated the relationship between preoperative systemic inflammatory markers (including SII, PNI, PLR and NLR) and the prognosis of osteosarcoma patients, and compared their accuracy.

We confirmed through univariate and multivariate Cox regression analysis that high preoperative SII and low PNI were independent risk factors for postoperative OS in osteosarcoma patients. The results of Kaplan-Meier survival analysis showed that the 5-year overall survival rate of the high SII group was significantly lower than that of the low SII group (27.7% vs. 87.0%), and the 5-year survival rate of the low PNI group was significantly lower than that of the high PNI group (23.1% vs. 83.6%), and the difference was statistically significant (*p* < 0.05). The main reasons for the analysis include: On the one hand, high SII level mostly represents high neutrophils and platelets and low lymphocyte level, suggesting that the body is in a tumor-promoting microenvironment, and its association with osteosarcoma progression can be attributed to the synergistic effects of its components: (1) Neutrophils, a key component of SII, accumulate in the osteosarcoma microenvironment via chemokines (e.g., CXCL8) secreted by tumor cells. They release reactive oxygen species (ROS) and matrix metalloproteinases (MMPs, such as MMP-9), which degrade the extracellular matrix (ECM) and bone matrix—critical for osteosarcoma’s local invasion into adjacent bone and soft tissues [19]; Additionally, neutrophils can form neutrophil extracellular traps (NETs), which trap circulating tumor cells, protect them from immune attack, and facilitate their extravasation during metastasis [19]. Neutrophils also secrete pro-inflammatory cytokines (e.g., IL-6, TNF-α) that activate NF-κB and STAT3 signaling pathways in osteosarcoma cells, promoting their proliferation and resistance to apoptosis [20]. (2) Elevated platelets in high SII reflect a pro-thrombotic state, which is closely linked to osteosarcoma metastasis. Platelets adhere to circulating tumor cells, forming “tumor-platelet aggregates” that shield tumor cells from natural killer (NK) cell-mediated lysis. They also release growth factors (e.g., TGF-β, PDGF) and angiogenic factors (e.g., VEGF), which stimulate endothelial cell proliferation and new blood vessel formation—supporting tumor growth and providing routes for distant metastasis [21]. In osteosarcoma, platelet-derived TGF-β can further induce epithelial-mesenchymal transition (EMT) in tumor cells, enhancing their migratory and invasive capacities [22]. (3) Lymphocytosis (low lymphocyte count) in high SII directly weakens adaptive immune monitoring. Cytotoxic T cells and NK cells are critical for recognizing and eliminating osteosarcoma cells; their reduction impairs tumor cell clearance [23]. Moreover, lymphocytes secrete anti-tumor cytokines (e.g., IFN-γ, IL-2) that inhibit tumor proliferation. A decline in lymphocytes disrupts this balance, allowing osteosarcoma cells to evade immune control and progress. On the other hand, the prognostic nutritional index (PNI) combines serum albumin and lymphocyte count, reflecting both nutritional status and immune competence. The decrease in PNI reflects that the body is in a state of malnutrition and immunosuppression, and its association with osteosarcoma progression is mediated by: (1) Hypoalbuminemia (low albumin) in low PNI indicates systemic malnutrition and metabolic stress, which exacerbate the osteosarcoma microenvironment. Albumin maintains vascular integrity; its reduction increases vascular permeability, facilitating tumor cell extravasation during metastasis [24, 25]. Additionally, hypoalbuminemia correlates with tumor-induced cachexia, where osteosarcoma cells compete for nutrients (e.g., glucose, amino acids), promoting their own proliferation at the expense of host tissues [26]. Low albumin also impairs the synthesis of immunoglobulins and complement proteins, weakening humoral immunity against tumor cells. (2) Similar to their role in SII, lymphocytes in PNI are critical for anti-osteosarcoma immunity. A reduction in lymphocytes (e.g., CD8 + T cells) impairs the recognition of tumor-associated antigens (e.g., HER2, p53 mutants) expressed by osteosarcoma cells, allowing unchecked growth. Lymphocytopenia also reduces the secretion of cytokines that recruit other immune cells (e.g., dendritic cells) to the tumor site, further dampening the anti-tumor immune response [27]. SII and PNI collectively reflect a “pro-tumor” phenotype: high SII indicates a pro-inflammatory, immune-suppressed state, while low PNI indicates compromised nutrition and immunity. Together, they create a microenvironment where osteosarcoma cells evade immune attack, invade surrounding tissues, and metastasize—ultimately worsening prognosis. For example, neutrophil-derived IL-6 (from high SII) can further suppress lymphocyte function (low PNI), forming a positive feedback loop that accelerates tumor progression.

In this study, the results of univariate and multivariate Cox regression analysis suggested that, in addition to SII and PNI, Enneking staging, local recurrence, and metastasis were also independent risk factors affecting the prognosis of osteosarcoma patients, which had important clinical significance. The Enneking staging system is a commonly used evaluation system in the clinical diagnosis and treatment of osteosarcoma. Its staging level is significantly positively correlated with tumor biological behavior and prognosis. With the increase of staging level, the degree of tumor histological differentiation decreases, the incidence of lung metastasis increases significantly, and the 5-year recurrence-free survival rate decreases significantly after surgery, and the patient’s prognosis becomes worse [28]. As for local recurrence or metastasis, it is reported that the probability of local recurrence in osteosarcoma patients after surgery is about 10–20%, and local recurrence often leads to poor prognosis for patients [29]. Na Linhao et al. [30] retrospectively analyzed the clinical data of 40 patients with osteosarcoma and confirmed that recurrence and metastasis are independent risk factors for the long-term prognosis of osteosarcoma. The 3-year survival rate of the recurrence and metastasis group was significantly lower than that of the non-recurrence and metastasis group (8.3% vs. 56.2%, χ^2^ = 17.494, *P* < 0.01), which is consistent with the results of this study.

Finally, based on the analysis results of the multivariate Cox regression model, this study screened out independent risk factors for the prognosis of osteosarcoma (Enneking stage, local recurrence, metastasis, SII, PNI), and constructed a nomogram based on this. After the construction was completed, the prognosis of osteosarcoma patients was predicted and evaluated with the help of this nomogram. To verify the predictive accuracy of the nomogram, this study used the C index (C index was 0.870 and the verification C index was 0.828) and calibration curve evaluation. The results showed that the nomogram had good discrimination and consistency, indicating that the nomogram showed good accuracy and practical value in predicting the prognosis of osteosarcoma patients. In addition, through ROC curve evaluation, we found that the AUC of nomogram prediction model is 0.976, which is significantly higher than that of SII, PNI and Enneking stage, indicating that this model is expected to be an effective tool to predict the prognosis of osteosarcoma patients. In addition, by analyzing the correlation between SII and PNI, we found that there was a significant negative correlation between SII and PNI (*r*=-0.410, *P* < 0.001), which confirmed the interdependence between systemic inflammation and nutritional immunity in patients with osteosarcoma. At the same time, we found that the patients with both high SII and low PNI (*n* = 37) had an extremely poor 5-year OS of only 14.8%, significantly lower than those with high SII alone (27.7%) or low PNI alone (23.1%) (log-rank *P* < 0.001). This suggests that the coexistence of pro-inflammatory and nutritional-immune dysfunction imposes a synergistic risk on prognosis, which also explains why the combination of SII and PNI outperforms individual indices in prognostic prediction, and emphasizes the necessity of evaluating both indices simultaneously. Mechanistically, this interaction may be mediated through the following pathways: on the one hand, pro-inflammatory cytokines (such as IL-6 and TNF-α) released by elevated neutrophils and platelets stimulate the liver to synthesize acute-phase proteins (e.g., C-reactive protein) and consume albumin, thus reducing the serum albumin levels, and at the same time, these cytokines further aggravate malnutrition by promoting muscle consumption and energy consumption [31]; On the other hand, low PNI indicates hypoalbuminemia and lymphopenia, which impairs the clearance of pro-inflammatory mediators and weakens the lymphocyte-mediated suppression of neutrophil/platelet activation [32]. This creates a vicious circle (malnutrition further amplifies the pro-inflammatory state (high SII), while inflammation worsens the nutritional depletion (low PNI)), which jointly accelerates the progression of tumors.

This study has certain limitations. First, as a single-center retrospective study, selection bias is difficult to avoid completely. Although Bootstrap repeated sampling verification was used, multicenter external verification is still needed. Second, this study did not include the evaluation of other inflammatory indicators, such as C-reactive protein, interleukin, etc. Furthermore, in some studies, the cutoff values of SII and PNI are not consistent, and most of them are considered to be certain, and the ideal cutoff value cannot be determined, which limits their practical application in clinical practice. Therefore, larger-scale prospective studies are still needed in the future to confirm our preliminary results. With further in-depth research in the later stage, SII and PNI can help medical staff provide options for surgery and treatment options.

In conclusion, this study confirmed that SII and PNI are not only independent predictors of osteosarcoma prognosis but also interact through the “inflammation-nutrition-immunity” axis, with their co-abnormality exacerbating poor outcomes. In addition, their detection has the advantages of being economical and convenient. The constructed nomogram, which integrates these two complementary indices with clinical characteristics, more accurately reflects the multi-faceted biological characteristics of osteosarcoma, providing a stronger basis for individualized treatment decisions.

## Data Availability

The datasets used and/or analyzed during the current study are available from the corresponding author on reasonable request.
